# Thinned Linear Optical Phased Array Design Through a Pareto-Optimal Synthesis Strategy

**DOI:** 10.3390/s25041096

**Published:** 2025-02-12

**Authors:** Xueqing Yang, Nicola Anselmi, Paolo Rocca

**Affiliations:** 1 ELEDIA Research Unit, CNIT—University of Trento, Via Sommarive 9, 38123 Trento, Italy; 2 ELEDIA Research Center (ELEDIA@UniTN—University of Trento), DICAM—Department of Civil, Environmental, and Mechanical Engineering, Via Mesiano 77, 38123 Trento, Italy; 3 ELEDIA Research Center (ELEDIA@XIDIAN—Xidian University), No. 2 South Taibai Road, Xi’an 710071, China

**Keywords:** optical phased arrays (OPAs), thinned arrays, waveguide grating antennas (WGAs), multi-objective optimization

## Abstract

The design of a thinned linear optical phased array (OPA) comprising a collection of waveguide grating antennas (WGAs) is addressed in this work. Given a fully populated linear OPA with antennas located in a uniform grid, the problem of selecting which elements have to be removed or retained is formulated as an optimization one. To this end, the definition of the optimal thinning architecture is produced through a multi-objective optimization strategy with the goal of minimizing the number of required antenna elements while maintaining a low sidelobe level and narrow beam width. A set of representative results is presented, also considering realistic WGA modeling to assess the capabilities and the potentialities of the proposed approach.

## 1. Introduction

Currently, optical phased arrays (OPAs) attract great attention and are being explored for utilization in a variety of applications such as light detection and ranging (LiDAR) [1,2], image projection [3], three-dimensional light printing [4], holographic displays [5], and free-space optical communications [6]. Indeed, thanks to the inherent advantages of electronic beam scanning, the capability of being compact, having high stability as well as resolution, and having a fast beam scanning speed make OPAs a very promising technology. Notably, unlike conventional one-dimensional electronic-based phased arrays, which can only achieve one-dimensional beam steering by adjusting the phase of each antenna element, one-dimensional OPAs composed of grating waveguide antennas (WGAs) exhibit the capability to achieve two-dimensional beam scanning [7,8], which is achieved primarily by adjusting wavelengths of the input light for steering along the WGA axis and tuning the phase of each element for scanning in the plane of the array axis. This adaptability has sparked significant research interest in one-dimensional OPAs.

Benefiting from the physical size advantages of optical antenna elements and advancements in manufacturing technology, classical one-dimensional OPAs can be readily designed as fully populated arrays with large sizes [2,9,10,11,12,13,14,15,16] to achieve a narrow beam width. However, this design approach results in OPAs comprising thousands of radiating elements, each requiring a transmit–receive module (TRM) which consists of an independently controlled amplifier and phase shifter, thus significantly increasing equipment complexity and costs.

In the field of the radio-frequency (RF) chain, unconventional one-dimensional arrays [17], which have already seen widespread application and have matured technologically, offer a solution to overcome this drawback. These unconventional arrays achieve cost reduction through two primary methods: reducing the number of antenna elements within specific apertures and sharing some TRMs among antenna groups. Sparse arrays [18] accomplish the former by arbitrarily distributing elements with larger spacing between them, while thinned arrays [19,20] achieve this by selectively removing a set of elements from lattice. Meanwhile, subarrays/clustered arrays [21] achieve the latter by grouping elements into several clusters. Inspired by advancements in these technologies, research on unconventional OPAs [5,8,22,23,24,25,26,27,28,29,30,31] has been incrementally explored in recent years. It is essential to acknowledge that while reducing the number of antenna elements, there is inevitably a trade-off: the performance of the radiation pattern is usually compromised compared to that of a fully populated array.

Among these investigations mentioned above, sparse linear OPAs have remained a focal point, as demonstrated in [8,23,24,25,28,29], where fewer antenna elements ensure a wide beam scanning range under the same aperture conditions as the fully populated arrays. However, this comes at the cost of compromised control over sidelobe level (SLL) and half-power beam width (HPBW). In order to deal with the higher SLL in sparse OPA, twice-optimization approaches are proposed in [26,27] where the array excitation undergoes a secondary optimization after the initial optimization of the OPA architecture. An alternative method [30] presented to suppress SLL sets two fitness functions of the maximum SLL when the beam steers. Nevertheless, despite these efforts, the HPBW remains less than the ideal level compared with the fully populated OPA. The application of cascaded phase shifters is discussed in [32] where three grouped phase shifters are used to control the beam scanning across 50 elements, offering a cost reduction. However, a similar issue with sparse OPAs persists: a high SLL and wide HPBW.

In such a framework, to the best of the authors’ knowledge, the novel design strategy of a one-dimensional thinned OPA that can minimize the number of elements while maintaining the radiation performance is first proposed in this paper. In detail, given the lattice of potential locations for the array elements, thinning the array involves turning off (removing) a set of antenna elements while turning on (retaining) the others to achieve a reduction in the number of elements. The “off” elements (removed) cannot contribute to the beam-forming network while only the “on” elements (retained) radiate signals. Considering the adverse effects on radiation patterns caused by diminishing antenna elements, an optimization method based on the multi-objective non-dominated sorting genetic algorithm is proposed here to ensure the quality of the pattern.

The novelty of this work over the existing works lies in the following: (i) the development of a multi-objective optimization algorithm for a thinning one-dimensional fully populated OPA, controlling the HPBW while reducing the number of antenna elements and achieving a lower SLL; (ii) the analysis of the thinned OPA performance considering realistic WGA models, thus enabling a two-dimensional beam steering capability.

The outline of this paper is given as follows. The OPA analysis problem is mathematically formulated in Section 2 where the analytic modeling of WGA is detailed as well. Section 3 presents a set of representative numerical results to validate the effectiveness of the proposed method along with a comparison with the state-of-the-art (SoA) research. Finally, conclusions are drawn in Section 4.

## 2. Mathematical Formulation

**Figure 1:**
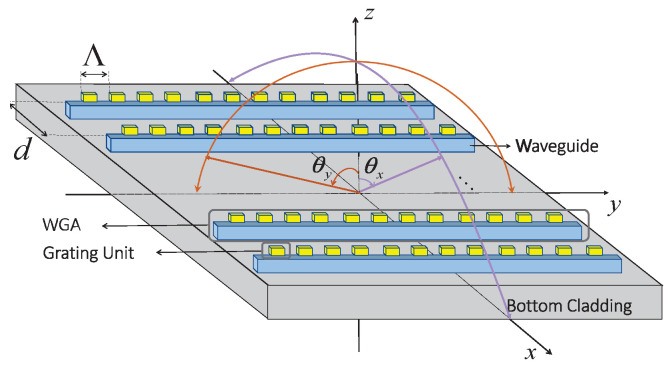
Sketch of typically linear, fully populated OPA.

Let us consider a linear, fully populated OPA (Figure 1) where *N* (N=2×G; assume an even number for analysis) waveguide grating antennas (WGAs) are equally spaced in a symmetrical distribution along the *x*-axis with the inter-element spacing *d*, and each WGA consists of *M* (M=2×O; assume an even number for analysis) grating units equally placed in a symmetrical distribution along the *y*-axis with the inter-element spacing Λ. Then, the far-field pattern radiated by this kind of OPA can be mathematically expressed as follows:

(1) $$\underline{E}\left(\theta_{x},\theta_{y}\right)≜{\underline{E}}_{g}\left(\theta_{y}\right)\times A\left(\theta_{x}\right)$$

where $A(\theta_{x})$ is the array factor which is determined by the architecture of the array, and ${\underline{E}}_{g}\left(\theta_{y}\right)$ is the active pattern of the *n*-th ($n\in N≜\left\{\pm 1,\ldots ,\pm G\right\}$) grating antenna which is presumed to be identical across all antennas [33], solely for the sake of simplifying notation without sacrificing generality, with $\theta_{x}$ and $\theta_{y}$ being the observation directions measured from the *z*-axis. It is noticeable that the orientation of the beam in $\theta_{x}$ is governed by the phase difference among the WGAs of the OPA, while the direction of the beam in $\theta_{y}$ is decided by the properties of the WGA and the wavelength of the input source. Additionally, $\theta_{y}$ can also be analyzed based on the phase difference in the grating units as an alternative method, which will be detailed later on.

**Figure 2:**
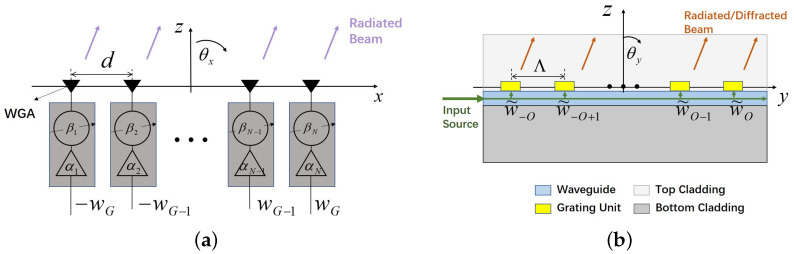
A sketch of the OPA in the (**a**) xz-plane (array view) and (**b**) yz-plane (antenna view).

Let us start from the mathematical analysis of the array factor $A(\theta_{x})$. For the sake of simplicity in the illustration, in Figure 2a, the long WGAs are represented by the small black triangles, showing the xz-plane of Figure 1 along with the addition of the excitation block. Each *n*-th WGA is complexly excited with $w_{n}$ ($w_{n}≜\alpha_{n}e^{j\beta_{n}}$), where $\alpha_{n}$ and $\beta_{n}$ are the corresponding amplitude and phase controlled by an amplifier and a phase shifter. Then, $A(\theta_{x})$ can be computed by

(2) $$A\left(\theta_{x}\right)≜\sum_{n\in N}w_{n}e^{jx_{n}k\sin\theta_{x}}$$

where k=2π/λ is the free-space wavenumber, with λ being the corresponding wavelength at the work frequency, and $x_{n}$ is the coordinates of the *n*-th antenna center:

(3) $$x_{n}=\left\{\begin{array}{c} (n+\frac{1}{2})d,\;n<0\\ (n-\frac{1}{2})d,\;n>0 \end{array}\right.$$

It is possible to analytically model the radiated far-filed ${\underline{E}}_{g}\left(\theta_{y}\right)$ of the WGA by considering the WGA as a fully populated array. The yz-plane of Figure 1 is reported in Figure 2b where the WGA along with the transmitted and radiated signal is illustrated in detail. In such a WGA, *M* (M=2×O; assume an even number) grating units are equally spaced along the *y*-axis, with the inter-element spacing Λ; then, the far-field pattern radiated by this “array” can be formulated by

(4) $${\underline{E}}_{g}\left(\theta_{y}\right)≜{\underline{E}}_{gu}\left(\theta_{y}\right)\times \tilde{A}\left(\theta_{y}\right)$$

where $\tilde{A}\left(\theta_{y}\right)$ is the quasi-array factor, and ${\underline{E}}_{gu}\left(\theta_{y}\right)$ is the active pattern of the *m*-th ($m\in M≜\left\{\pm 1,\ldots ,\pm O\right\}$) grating unit which is assumed to be the quasi-isotropic element (i.e., ${\underline{E}}_{gu}\left(\theta_{y}\right)=1$) [34,35]. And, therefore, ${\underline{E}}_{g}\left(\theta_{y}\right)$ can be rewritten as ${\underline{E}}_{g}\left(\theta_{y}\right)≜\tilde{A}\left(\theta_{y}\right)$. When the input source propagates along the waveguide, each *m*-th grating unit is complexly “excited” with ${\tilde{w}}_{m}$ being ${\tilde{w}}_{m}≜{\tilde{\alpha }}_{m}e^{j{\tilde{\beta }}_{m}}$, where ${\tilde{\alpha }}_{m}$ and ${\tilde{\beta }}_{m}$ are the corresponding “amplitude” and “phase”. The “amplitude” ${\tilde{\alpha }}_{m}$ indicates the magnitude of the electric field of the *m*-th grating unit

(5) $${\tilde{\alpha }}_{m}=E_{0}e^{-\kappa (y_{m}-y_{1})}$$

where $E_{0}$ is the magnitude of the electric field at the beginning of the grating unit, $y_{m}$ is the the coordinates of the *m*-th grating unit center, and κ is the grating strength. The “phase” ${\tilde{\beta }}_{m}$ of the *m*-th grating unit is defined as

(6) $${\tilde{\beta }}_{m}≜-{\tilde{\beta }}_{-1}\left(y_{m}-y_{1}\right)$$

where ${\tilde{\beta }}_{-1}$ is the negative first-order spacial harmonic propagation constant (harmonics of other orders are evanescent waves not able to radiate) [35]. Then, the far field radiated by the WGA ${\underline{E}}_{g}\left(\theta_{y}\right)$ can be mathematically expressed as

(7) $${\underline{E}}_{g}\left(\theta_{y}\right)=\tilde{A}\left(\theta_{y}\right)≜\sum_{m\in M}{\tilde{w}}_{m}e^{jk_{h}y_{m}\sin\theta_{y}}$$

where $k_{h}=2\pi n_{h}/\lambda$ is the wavenumber, with λ and $n_{h}$ being the corresponding wavelength at the work frequency and the refractive index of the top cladding, respectively, and $y_{m}$ is the coordinates of the *m*-th grating unit center:

(8) $$y_{m}=\left\{\begin{array}{c} (m+\frac{1}{2})\Lambda ,\;m<0\\ (m-\frac{1}{2})\Lambda ,\;m>0 \end{array}\right.$$

**Figure 3:**
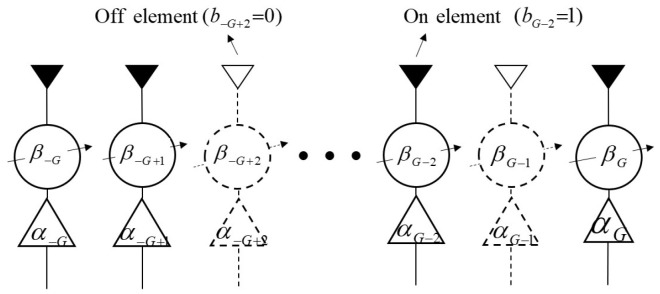
A sketch of the thinning OPA architecture.

Thinning an OPA, where a set of antenna elements is turned off while the others are turned on (Figure 3), can be mathematically formulated by introducing the Boolean vector $B=\left\{b_{n};n\in N\right\}$ [36], and $b_{n}\in \left\{0,1\right\}$ is a binary entry used to identify which elements are off/on. Therefore, the excitation of thinning array is $w_{n}\to w_{n}b_{n}$, and the array factor should be

(9) $$A\left(\theta_{x}\right)≜\sum_{n\in N}b_{n}w_{n}e^{jx_{n}k\sin\theta_{x}}$$

In order to reduce the complexity of the OPA architecture, the *thinning array synthesis problem* (TASP) at hand can be formulated as follows.

Given a fully populated linear PA with *N* radiating elements located in a uniform grid with the spacing *d*, we find the optimal thinning configuration of the array, $B^{\mathrm{opt}}$, using the corresponding excitation weight $w^{\mathrm{opt}}$ ($w^{\mathrm{opt}}=\left\{w_{n}b_{n}^{opt};n\in N\right\}$), so that the *Q*-size features of the array and the radiated power pattern $F\left\{P(\theta_{x})|B\right\}=\left\{F_{q}\left\{P(\theta_{x})|B\right\};q=1,\ldots ,Q\right\}$ fit the user-defined targets $F^{\mathrm{tar}}=\left\{F_{q}^{tar};q=1,\ldots ,Q\right\}$, with $F_{q}^{tar}$ being the *q*-th requirement, by minimizing the fitness functions Γ(B) ($\Gamma \left(B\right)≜\left\{\Gamma_{q}\left(B\right);q=1,\ldots ,Q\right\}$), where

(10) $$\Gamma_{q}\left(B\right)=\int_{-\frac{\pi }{2}}^{\frac{\pi }{2}}\frac{{\left|F_{q}\left\{P(\theta_{x})|B\right\}-F_{q}^{tar}\right|}^{2}}{{\left|F_{q}^{tar}\right|}^{2}}\times H\left\{F_{q}\left\{P(\theta_{x})|B\right\}-F_{q}^{tar}\right\}d\theta_{x}\left(q=1,\ldots ,Q\right)$$

with $P\left(\theta_{x}\right)={\left|A(\theta_{x})\right|}^{2}$ and H(·) being the Heaviside function.

To tackle the TASP described above, a synthesis strategy based on the multi-objective algorithm (MOA) is presented. From a mathematical perspective, the multi-objective problem manifests as the Pareto front (PF) that offers a collection of “optimal” (i.e., non-dominated) trade-off solutions which align with the *Q*-size requirements defined by the user (i.e., satisfying $F^{\mathrm{tar}}$). Notably, these requirements usually conflict with each other. The non-dominated sorting genetic algorithm (NSGA-III) [37], which starts with a set of possible solutions and moves toward the “best” one through an evolutionary process, is applied to solve the formulated problem. The goal is to optimize the array architecture (i.e., reducing τ, $\tau =\sum_{n=1}^{N}b_{n}$), while ensuring the quality of the radiation power pattern. Here, the metrics used for evaluation of the pattern performance are the SLL ($SLL≜\max_{\theta_{x}\in \Omega_{SLL}}\left\{-10\times \log\left[P(\theta_{x})\right]\right\}$, where $\Omega_{SLL}$ and $P(\theta_{x})$ are the SLL region and the normalized power pattern) and the HPBW ($\Theta_{h}≜\left|\theta_{x_{r}}-\theta_{x_{l}}\right|,P\left(\theta_{x_{r}}\right)=P\left(\theta_{x_{l}}\right)=\frac{\max_{\theta_{x}\in \Omega_{m}}\left[P(\theta_{x})\right]}{2}$, where $\Omega_{m}$ is the mainlobe region). The details of the optimization procedure are as follows.

- *Step 0—Desired Thinned Array Definition*. Based on the number of candidate array elements, *N*, the inter-element spacing *d*, and the array excitations w, the *Q* features $F^{\mathrm{tar}}=\left\{F_{q}^{tar};q=1,\ldots ,Q\right\}$ are defined and to be matched in (10). Three user-defined requirements (i.e., Q=3) are reported here: (1) the number of active elements $\tau^{tar}$ (i.e, $F_{q}^{tar}\rfloor_{q=1}=\tau^{tar}$); (2) the SLL (i.e., $F_{q}^{tar}\rfloor_{q=2}=SLL^{tar}$); and (3) the HPBW (i.e., $F_{q}^{tar}\rfloor_{q=3}=\Theta_{h}^{tar}$).
- *Step 1—Initialization*. Under the iteration index i=0, the alphabet/population $B_{i}\rfloor_{i=0}$ with *K* words/individuals, $B_{i}\rfloor_{i=0}≜\left\{B_{i}^{\left(k\right)}\rfloor_{i=0};k=1,\ldots ,K\right\}$, is firstly generated for sampling the solution-space. Then, for each *k*-th term, we calculate the corresponding features $F_{i}^{\left(k\right)}\rfloor_{i=0}$ ($F_{i}^{\left(k\right)}\rfloor_{i=0}≜\left\{F_{q,(i)}^{\left(k\right)}\rfloor_{i=0};q=1,\ldots ,Q\right\}$) of the radiated pattern and fitness functions $\Gamma^{\left(k\right)}\rfloor_{i=0}$ ($\Gamma^{\left(k\right)}\rfloor_{i=0}≜\left\{\Gamma_{q,(i)}^{\left(k\right)}\rfloor_{i=0};q=1,\ldots ,Q\right\}$) based on the corresponding Boolean vector $B_{i}^{\left(k\right)}\rfloor_{i=0}$.
- *Step 2—Pareto Ranking*. The *R*-level PFs are defined by ranking the individuals according to the Pareto dominance rule (PDR): for each (*k*, *c*)-th (*k*, c=1,…,K, c≠k) couple of words, ($B^{\left(k\right)}$, $B^{\left(c\right)}$), if $\Gamma_{q}\left(B^{\left(k\right)}\right)\leq \Gamma_{q}\left(B^{\left(c\right)}\right)$, (q=1,…,Q) and there exists a *g*-th ($g\in \left[1,\ldots ,Q\right]$) fitness function such that $\Gamma_{g}\left(B^{\left(k\right)}\right)<\Gamma_{g}\left(B^{\left(c\right)}\right)$, then $B^{\left(k\right)}$ is the non-dominated solution. By selecting the set of non-dominated solutions at the (r−1) level based on the PDR, the composition of the *r*-th level of the PF is formed, with the first level (r=1) of the PF being generated by applying the PDR to the entire population $B_{i}$. A pre-allocated reference set mechanism is used to choose better diverse solutions within the same *r*-th (r=1,…,R) level [37].
- *Step 3—Population Update*. By updating the iteration index (i←i+1), a new temporary population $T_{i}$ with *K* individuals is generated through a sequence of crossovers and mutations according to the NSGA-III evolutionary strategy. We calculate the corresponding *K*-size fitness function $\Gamma^{\left(k\right)}$ ($\Gamma^{\left(k\right)}≜\left\{\Gamma_{i}^{\left(k\right)};k=1,\ldots ,K\right\}$) and rank all the individuals of $B_{i-1}$ and $T_{i}$ according to Step 2 to form a new population, $B_{i}$, with the first *K* ranked individuals.
- *Step 4—Termination Criteria*. we repeat Step 2 and Step 3 until the iteration index *i* reaches the user-defined maximum value *I* (i.e., i=I).
- *Step 5—Optimal Thinning Computation*. A set of (*S*-sized) optimal trade-off solutions ($B^{PF}$, $B^{PF}≜\left\{B^{\left(s\right)};s=1,\ldots ,S\right\}$) is generated after convergence. Then, the optimal solution $B^{\mathrm{opt}}$ is selected based on the minimum Manhattan distance (MMD) method [38], defined as
  (11) $$B^{\mathrm{opt}}=\arg\left\{\min_{s=1,\ldots ,S}\left[{∥\Gamma^{\left(s\right)}-\Gamma^{\left(ideal\right)}∥}_{1}\right]\right\}$$
  where ${∥\cdot ∥}_{1}$ is the L1 norm and the *q*-th (q=1,…,Q) elements of $\Gamma^{\left(s\right)}$ ($\Gamma^{\left(s\right)}≜\left\{{\hat{\Gamma }}_{q}^{\left(s\right)};q=1,\ldots ,Q\right\}$) and $\Gamma^{\left(ideal\right)}$ ($\Gamma^{\left(ideal\right)}≜\left\{\Gamma_{q}^{\left(ideal\right)};q=1,\ldots ,Q\right\}$) are shown as follows:
  (12) $${\hat{\Gamma }}_{q}^{\left(s\right)}=\frac{\Gamma_{q}\left(B^{\left(s\right)}\right)}{\max_{s=1,\ldots ,S}\left[\Gamma_{q}\left(B^{\left(s\right)}\right)\right]-\min_{s=1,\ldots ,S}\left[\Gamma_{q}\left(B^{\left(s\right)}\right)\right]}$$
  (13) $$\Gamma_{q}^{\left(ideal\right)}=\min_{s=1,\ldots ,S}\left[{\hat{\Gamma }}_{q}\left(B^{\left(s\right)}\right)\right]$$

## 3. Numerical Results

In this section, the selected numerical results are reported to evaluate the effectiveness of the proposed method.

This first example is aimed at validating the MOA-based method for the thinning OPA, and it deals with a linear array composed of N=256 isotropic elements ($w_{n}=1$, $n\in \left\{\pm 1,\ldots ,\pm 128\right\}$) distributed in a uniform (d=1.29λ) lattice [13]. The target number of “on” WGAs $\tau^{tar}$ is set to be half of *N* (i.e., $F_{q}^{tar}\rfloor_{q=1}=128$) to reduce the retained number of elements by 50%. The other two targets include the maximum SLL in the field of view (FOV) $SLL^{tar}$ being −20 [dB] (i.e., $F_{q}^{tar}\rfloor_{q=2}=-20$) and the HPBW $\Theta_{h}^{tar}$ being as close as possible to that of the fully populated array (i.e., $F_{q}^{tar}\rfloor_{q=3}=0.154$ [deg]). Since the inter-element spacing *d* is larger than λ/2, the FOV is limited (i.e., FOV is 45.60 [deg] when d=1.29λ) [33]. The MOA parameters are set as follows: B=N (size of alphabet); I=1000 (maximum number of iterations); the cross-over rate $c_{R}=1.0$; the cross-over distribution index $c_{I}=15.0$; the mutation rate $m_{R}=1/N$; and the distribution index for polynomial mutation $m_{I}=20.0$.

**Figure 4:**
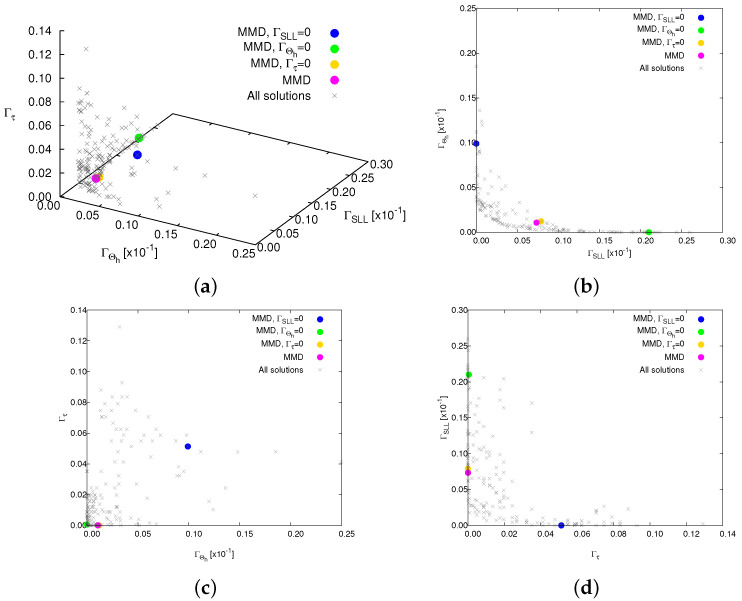
*Numerical validation* (N=256; $\tau^{tar}=128$; $SLL^{tar}=-20$ [dB]; $\Theta_{h}^{tar}=0.154$ [deg]; P=N; I=1000)—a plot of the Pareto front in the (**a**) ($\Gamma_{\Theta_{h}},\Gamma_{SLL},\Gamma_{\tau }$) plane, (**b**) ($\Gamma_{SLL},\Gamma_{\Theta_{h}}$) plane, (**c**) ($\Gamma_{\Theta_{h}},\Gamma_{\tau }$) plane, and (**d**) ($\Gamma_{\tau },\Gamma_{SLL}$) plane. The grey crosses denote all output solutions, and the magenta-colored circle denotes the “optimal solution” selected based on the MMD method from all solutions; the blue, green, and yellow circles indicate the optimal solutions selected based on the MMD from solutions satisfying the objectives of the SLL (i.e., $\Gamma_{SLL}=0$), the HPBW (i.e., $\Gamma_{HPBW}=0$), and τ (i.e., $\Gamma_{\tau }=0$), respectively.

**Figure 5:**
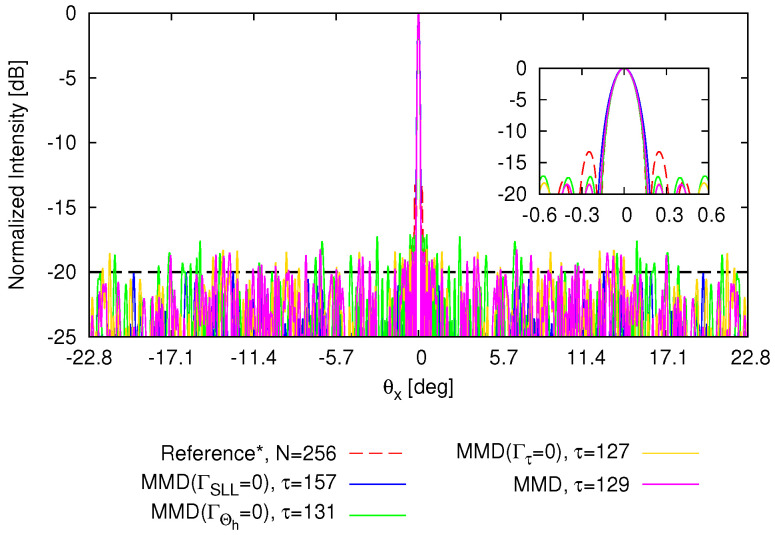
*Numerical validation* (N=256; $\tau^{tar}=128$; $SLL^{tar}=-20$ [dB]; $\Theta_{h}^{tar}=0.154$ [deg]; P=N; I=1000)—a plot of normalized power patterns radiated by the thinned OPAs.

**Figure 6:**
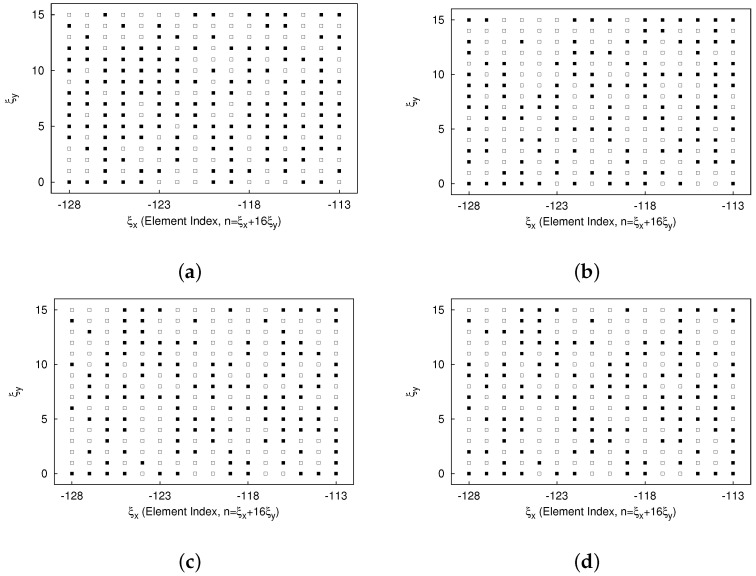
*Numerical validation* (N=256; $\tau^{tar}=128$; $SLL^{tar}=-20$ [dB]; $\Theta_{h}^{tar}=0.154$ [deg]; P=N; I=1000)—a plot of the thinned layout of the selected solutions based on the MMD from (**a**) solutions with $\Gamma_{SLL}=0$, (**b**) solutions with $\Gamma_{HPBW}=0$, (**c**) solutions with $\Gamma_{\tau }=0$, and (**d**) all solutions.

Figure 4 shows the PF, comprising 172 solutions (grey crosses) along with four emphasized solutions: the magenta-colored circle denotes the “optimal solution” selected based on the MMD method from all solutions; the blue, green, and yellow circles indicate the optimal solutions selected based on MMD from solutions satisfying the objectives of the SLL (i.e., $\Gamma_{SLL}=0$), the HPBW (i.e., $\Gamma_{HPBW}=0$), and τ (i.e., $\Gamma_{\tau }=0$), respectively. The corresponding normalized power patterns in comparison with the fully populated array are given in Figure 5, while the thinned layouts corresponding to these four selected solutions are reported in Figure 6. The pattern features are described in detailed in Table 1 as well. From these results, it can be seen that the solution based on MMD closely approach the three user-defined targets. Utilizing nearly half the array elements ΔN=49.61 [%] (ΔN≜(N−τ)/N×100), this thinned OPA achieves lower SLL ${△}_{SLL}^{Slected-ref}SLL=-5.03$ [dB], albeit with a slightly wider HPBW ${△}_{\Theta_{h}}^{MMD-ref}=0.005$ [deg].

**Figure 7:**
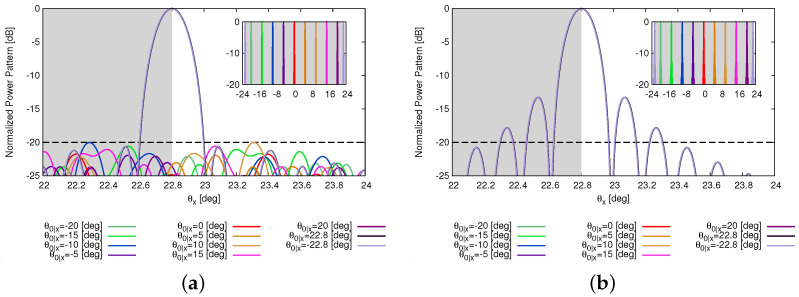
*Numerical validation* (N=256; $\tau^{tar}=128$; $SLL^{tar}=-20$ [dB]; $\Theta_{h}^{tar}=0.154$ [deg]; P=N; I=1000)—a plot of the beam-steered patterns radiated by (**a**) the thinned layouts in Figure 6a and (**b**) the fully populated array. The grey rectangle shows the FOV (45.60 [deg]) of the arrays.

**Figure 8:**
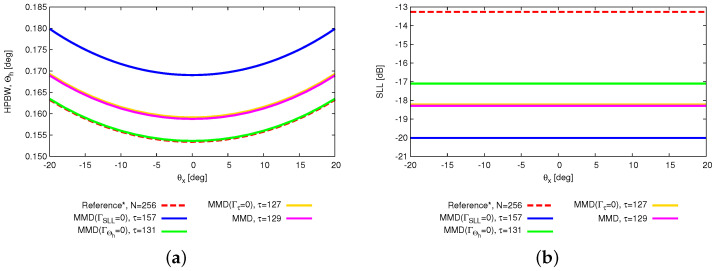
*Numerical validation* (N=256; $\tau^{tar}=128$; $SLL^{tar}=-20$ [dB]; $\Theta_{h}^{tar}=0.154$ [deg]; P=N; I=1000)—a plot of the behavior of the (**a**) SLL and (**b**) HPBW when the beam steers from −20 [deg] to 20 [deg].

Among the solutions satisfying one target, there exists disparity in the other two target values. However, the ultimate objective of reducing “on” elements while ensuring the quality of the power pattern is achieved. This also reinforces the notion of “no free lunch” when addressing multiple conflicting requirements. Toward this end, users can select the “optimal” solution according to their specific requirements. Then, the capability of beam steering in the $\theta_{x}$ direction by the thinned OPA is analyzed, as shown in Figure 7. The beam is steered from −22.8 [deg] (i.e., $\theta_{0|x}=-22.8$ [deg]) to 22.8 [deg] (i.e., $\theta_{0|x}=22.8$ [deg]). It can be observed that the optimized array can achieve the same beam scanning range as the reference array. The behaviors of the SLL and HPBW when the OPAs steer are reported in Figure 8. The SLL keep consistent when beam steers.

**Figure 9:**
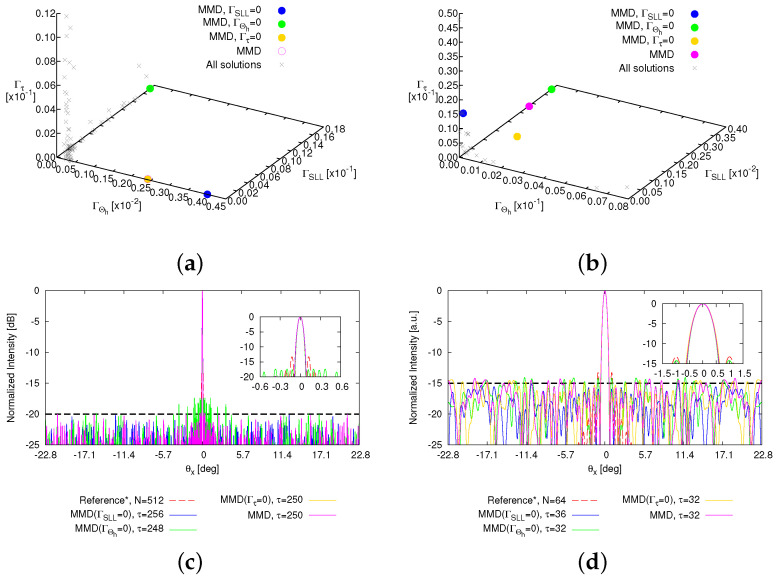
*Numerical assessment* (*P* = *N*; *I* = 1000)—a plot of (**a**,**b**) the PFs and (**c**,**d**) the compared normalized patterns. (**a**,**c**) We set *N* = 512; $\tau^{tar}=256$; $SLL^{tar}=-20$ [dB]; and $\Theta_{h}^{tar}=0.077$ [deg]. (**b**,**d**) We set N=64; $\tau^{tar}=32$; $SLL^{tar}=-15$ [dB]; and $\Theta_{h}^{tar}=0.164$ [deg].

To further assess the reliability of the proposed method, the second set of test cases considers the same target thinning percentage $\Delta N^{tar}=50$ [%] as the first test case but increases and decreases the number of elements of the reference fully populated OPA to N=512 ($\tau^{tar}=256$) and N=64 ($\tau^{tar}=32$), respectively. Moreover, as the inter-element spacing *d* remains constant (i.e., d=1.29λ), the FOV stays constant, while the array aperture changes, resulting in the HPBW narrowing to $\Theta_{h}=0.077$ [deg] and widening to $\Theta_{h}=0.614$ [deg], respectively. The same maximum SLL within the FOV is set to be $SLL^{tar}=-20$ [dB] firstly. In all cases, the MOA parameters are set to be the same as the first case. The corresponding PFs and compared patterns are reported in Figure 9, while the details of these pattern features are summarized in Table 2. When optimizing the array with N=512 elements, the number of active elements are thinned by more than half, namely ΔN≥50 [%], while power patterns maintain good performance. More specifically, the maximum SLL can be suppressed to ${△}_{SLL}^{Slected-ref}SLL=6.83$ [dB] with τ=256 elements while the HPBW is marginally wider at ${△}_{\Theta_{h}}^{MMD-ref}=0.004$ [deg]. When the HPBW meets or even exceeds the objectives (i.e., $\Theta_{h}^{tar}=0.077$ [deg]), ${△}_{\Theta_{h}}^{MMD-tar}=-0.001$ [deg]; although the SLL is higher than the targets ${△}_{SLL}^{Slected-tar}SLL=2.62$ [dB], it is significantly lower compared to the fully populated array ${△}_{SLL}^{Slected-ref}SLL=-4.12$ [dB], and the thinning rate remarkably reaches ΔN=51.56 [%]. However, when the population size is small (i.e., B=64) for optimizing the OPA with N=64 elements, thinning the OPA poses challenges in maintaining the quality of the radiation pattern; even after I=1000 iterations, the solution satisfying the objective of either SLL (i.e., $\Gamma_{SLL}=0$) or of the HPBW (i.e., $\Gamma_{HPBW}=0$) is not found (Table 2). As for the sole selected “optimal” solution, its radiated pattern exhibits a high SLL and wide HPBW (i.e., ${△}_{SLL}^{Slected-ref}SLL=-0.02$ [dB]; ${△}_{\Theta_{h}}^{MMD-ref}=0.073$ [deg]). To tackle this issue, a potential approach involves moderating the optimization complexity, such as by adjusting the target SLL $SLL^{tar}$ to −15 [dB] (i.e., $F_{q}^{tar}\rfloor_{q=2}=-15$). Then, the simulation for the thinning OPA with N=64 elements is conducted with three objectives: (1) $F_{q}^{tar}\rfloor_{q=1}=128$; (2) $F_{q}^{tar}\rfloor_{q=2}=-15$; and (3) $F_{q}^{tar}\rfloor_{q=3}=0.614$. The results are given in Figure 9d and Table 2. As expected, the selected “optimal” solutions can achieve patterns close to user-defined requirements. Therefore, given the stochastic nature of the optimization algorithm NSGA-III, it is crucial to set targets that are reasonable and aligned with the design context to ensure an effective and reliable achievement of the optimization goals [38].

The third set of test cases are devoted to give a comparison with SoA works. In this part, three SoA references are selected, as these works include the analysis of OPAs with isotropic elements: two works relate to fully populated OPAs [2,13], and one relates to a sparse OPA [31]. Table 3 summarizes the comparison between SoAs and the results optimized in this work, where the optimized thinned array is here selected based on the MMD among all the solutions. The other specific parameters for these three optimization are as follows: (1) For the thinning OPA in [13], N=256; d=1.29λ; $F_{q}^{tar}\rfloor_{q=1}=128$; $F_{q}^{tar}\rfloor_{q=2}=-20$; and $F_{q}^{tar}\rfloor_{q=3}=0.154$. (2) For the thinning OPA in [2], N=512; d=1.06λ; $F_{q}^{tar}\rfloor_{q=1}=256$; $F_{q}^{tar}\rfloor_{q=2}=-20$; and $F_{q}^{tar}\rfloor_{q=3}=0.095$. (3) For comparing the OPAs in [31], N=102; d=3.17λ; $F_{q}^{tar}\rfloor_{q=1}=64$; $F_{q}^{tar}\rfloor_{q=2}=-20$; and $F_{q}^{tar}\rfloor_{q=3}=0.238$. These comparative results demonstrate the feasibility and advantages of the method proposed in this paper.

**Figure 10:**
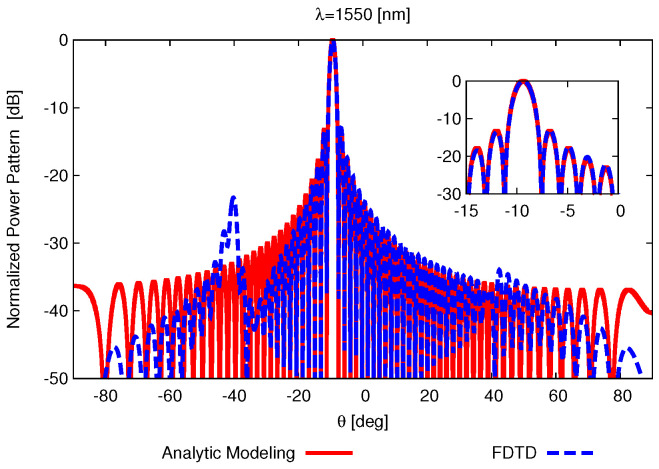
*Robustness analysis* (non-ideal radiating elements; λ=1550 [nm]; $\theta_{0\left|y\right.}{|}_{\lambda =1550}=$−9.36 [deg])—a plot of the compared normalized power pattern cut along the $\theta_{x}=0$ plane between FDTD and analytic modeling.

The final case aims to offer some insights when the array consists of realistic radiating elements (i.e., long-waveguide grating antennas). The implementation here is conducted in three primary steps: (1) utilizing the finite difference time domain (FDTD) to numerically compute the far-field radiation characteristics of a single WGA [39]; (2) extracting the propagation constant ${\tilde{\beta }}_{-1}$ [35] and grating strength κ from the FDTD results and theoretically calculating ${\underline{E}}_{g}\left(\theta_{y}\right)$ (7), with κ being obtained based on (5) where ${\tilde{\alpha }}_{m}$ is measured by placing monitors at the center of the etches during FDTD simulations; and (3) deriving the power pattern radiated by the OPA (4). The 50 [μm]-long dual-layer WGA [40], which is consisted of M=70 grating units with the spacing Λ=700 [nm], is considered here to form an OPA with N=256 and the element spacing d=2 [μm] working at f=193.55 [THz] (i.e., λ=1550 [nm]). Firstly, the far-field projection by the WGA is simulated with FDTD, and the corresponding normalized power pattern cut along the $\theta_{x}=0$ plane is shown in Figure 10 with the main beam at $\theta_{0\left|y\right.}{|}_{\lambda =1550}=-9.36$ [deg].

**Figure 11:**
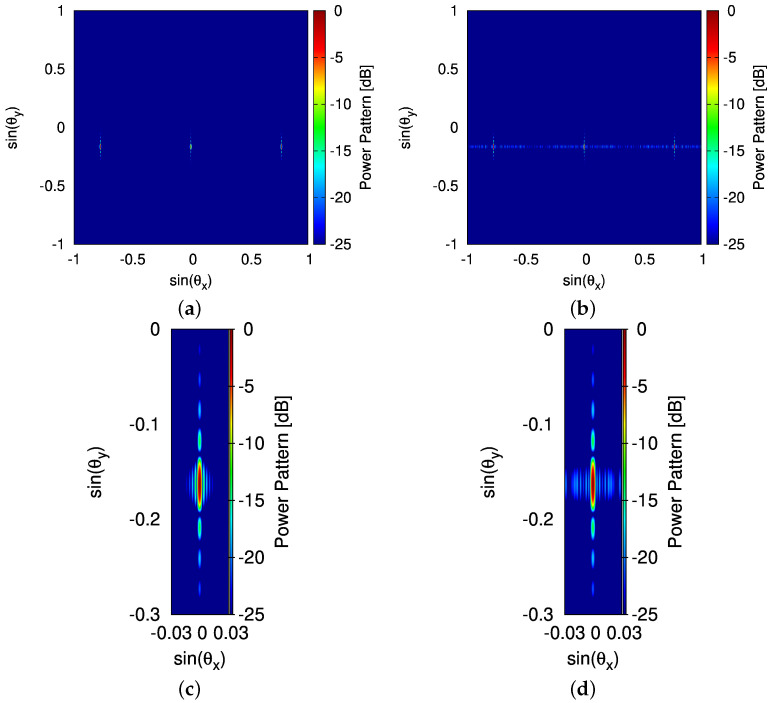
*Robustness analysis* (non-ideal radiating elements; λ=1550 [nm]; $\theta_{0\left|x\right.}{|}_{\lambda =1550}=0$ [deg]; $\theta_{0\left|y\right.}{|}_{\lambda =1550}=-9.36$ [deg])—a plot of the normalized power pattern radiated by setting (**a**,**c**) a fully populated OPA and (**b**,**d**) a thinned OPA in Figure 6d; (**c**,**d**) are magnified sections of (**a**,**b**), respectively.

Then, the propagation constant ${\tilde{\beta }}_{-1}$ and grating strength κ extracted from the FDTD results, which are ${\tilde{\beta }}_{-1}{|}_{\lambda =1550}=-6.59\times 10^{-4}$ [nm^−1^] and $\kappa {|}_{\lambda =1550}=1.85\times 10^{-7}$ [nm^−1^], are used for analytic modeling, and the results are compared with the FDTD simulation (Figure 10). It is found that the radiation patterns are highly consistent, except for an SLL of less than −20 [dB] near −40 [deg] , which is attributed to perturbations caused by the first few grating units [35]. Finally, integrated with the far-field pattern of the real element, the normalized power patterns radiated by the fully populated OPA and thinned OPA (Figure 6d) are compared in Figure 11. The three equal-intensity spots are shown due to the presence of the other two grating lobes (at $\theta_{0|x}=\pm 50.81$ [deg]) as d>λ/2.

**Figure 12:**
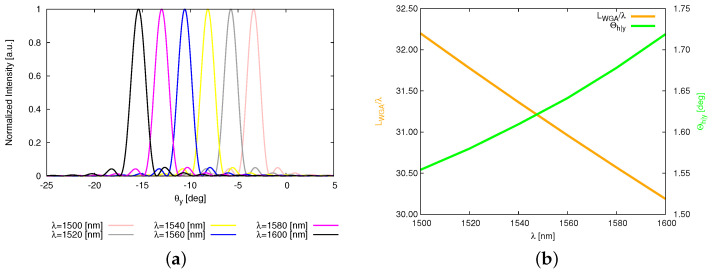
*Robustness analysis* (non-ideal radiating elements, tuning the input λ from 1500 [nm] to 1600 [nm] increasing 20 [nm])—a plot of (**a**) the normalized electric intensity cut along the $\theta_{x}=0$ plane and (**b**) $\Theta_{h\left|y\right.}$ variation with λ.

**Figure 13:**
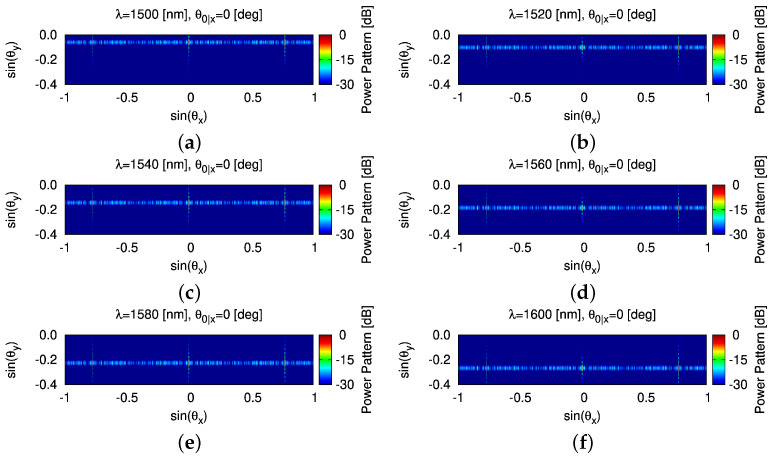
*Robustness analysis* (non-ideal radiating elements, $\theta_{0\left|x\right.}=0$ [deg])—a plot of the normalized power patterns with the beam steered and the thinned OPA in Figure 6d radiating (**a**) $\theta_{0\left|y\right.}{|}_{\lambda =1500}=-3.36$ [deg]; (**b**) $\theta_{0\left|y\right.}{|}_{\lambda =1520}=-5.74$ [deg]; (**c**) $\theta_{0\left|y\right.}{|}_{\lambda =1540}=-8.16$ [deg]; (**d**) $\theta_{0\left|y\right.}{|}_{\lambda =1560}=-10.56$ [deg]; (**e**) $\theta_{0\left|y\right.}{|}_{\lambda =1580}=-12.98$ [deg]; and (**f**) $\theta_{0\left|y\right.}{|}_{\lambda =1600}=-15.43$ [deg].

**Figure 14:**
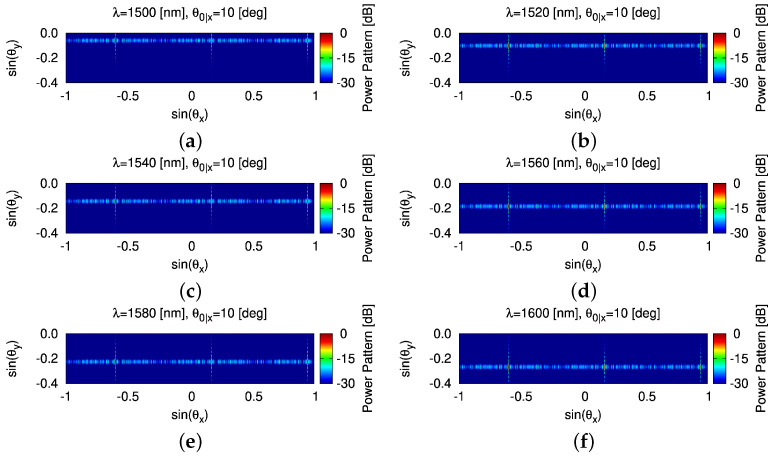
*Robustness analysis* (non-ideal radiating elements, $\theta_{0\left|x\right.}=10$ [deg])—a plot of the normalized power patterns with the beam steered and the thinned OPA in Figure 6d radiating (**a**) $\theta_{0\left|y\right.}{|}_{\lambda =1500}=-3.36$ [deg], (**b**) $\theta_{0\left|y\right.}{|}_{\lambda =1520}=-5.74$ [deg], (**c**) $\theta_{0\left|y\right.}{|}_{\lambda =1540}=-8.16$ [deg], (**d**) $\theta_{0\left|y\right.}{|}_{\lambda =1560}=-10.56$ [deg], (**e**) $\theta_{0\left|y\right.}{|}_{\lambda =1580}=-12.98$ [deg], and (**f**) $\theta_{0\left|y\right.}{|}_{\lambda =1600}=-15.43$ [deg].

It is worth noting that one-dimensional OPA enables a two-dimensional beam scanning: (1) steering along $\theta_{y}$ (WGA axis) by tuning λ and (2) steering along $\theta_{x}$ (array axis) by adjusting the phase of WGAs. Therefore, let us change the wavelength λ of the input light starting from 1500 [nm], increasing 20 [nm] each time, up to 1600 [nm]. It is observed that the beam steers from $\theta_{0\left|y\right.}{|}_{\lambda =1500}=-3.36$ [deg] to $\theta_{0\left|y\right.}{|}_{\lambda =1600}=-15.43$ [deg], achieving a steering range in $\theta_{y}$ around 12 [deg] (Figure 12a). The HPBW in the WGA axis $\Theta_{h\left|y\right.}$ increases as the input wavelength increases (Figure 12b). The corresponding ${\tilde{\beta }}_{-1}$ and κ extracted to generate analytic modeling results are shown in Table 4 where the pattern features are detailed as well. Finally, the two beam steered patterns radiated by the thinned OPA (Figure 6d) are summarized in Figure 13 and Figure 14 where the beam in the $\theta_{x}$ direction are at 0 [deg] (i.e., $\theta_{0\left|x\right.}=0$ [deg]) (Figure 13) and 10 [deg] (i.e., $\theta_{0\left|x\right.}=10$ [deg]) (Figure 14), and the beam in the $\theta_{y}$ direction shifts from $\theta_{0\left|y\right.}{|}_{\lambda =1500}=-3.36$ [deg] to $\theta_{0\left|y\right.}{|}_{\lambda =1600}=-15.43$ [deg].

## 4. Conclusions

The design of the thinned one-dimensional OPA comprising grating waveguide antennas (WGAs) was addressed by employing a multi-objective optimization strategy. This approach ensured the reduction in the antenna elements while simultaneously guaranteeing the performance of the radiated pattern.

To the best of the authors’ knowledge, the main innovative contributions of this work compared to existing SoA are as follows:

1. The exploitation of a Pareto-optimal design strategy for the thinning of a linear OPA when considering multiple conflicting requirements such as SLL suppression, HPBW constrains, and the minimization of the number of elements;
2. The use of realistic WGA models during the analysis of the thinned OPA in order to investigate the two-dimensional beam steering capability.

Future research activities, outside the scope and objective of the current work, will be aimed at extending the proposed approach to planar and conformal arrays.

## Figures and Tables

**Table 1:** *Numerical validation* (N=256; $\tau^{tar}=128$; $SLL^{tar}=-20$ [dB]; $\Theta_{h}^{tar}=0.154$ [deg]; P=N; I=1000)—pattern features.

Solution	τ	SLL	$\Theta_{h}$	*L*	Δ*N*
		[dB]	[deg]	[*λ*]	[%]
*Reference*	256	−13.26	0.154	329.03	0
MMD, $\Gamma_{SLL}=0$	157	−20.01	0.169	329.03	38.67
MMD, $\Gamma_{\Theta_{h}}=0$	131	−17.10	0.154	329.03	48.84
MMD, $\Gamma_{\tau }=0$	127	−18.22	0.159	329.03	50.39
MMD	129	−18.29	0.159	329.03	49.61

**Table 2:** *Numerical validation* (P=N; I=1000)—pattern features.

*N*	${\mathrm{SLL}}^{\mathrm{tar}}$	Solution	τ	SLL	$\Theta_{h}$	*L*	ΔN
	[dB]			[dB]	[deg]	[λ]	[%]
512	-	Fully populated	512	−13.26	0.077	659.35	0
512	−20	MMD, $\Gamma_{SLL}=0$	256	−20.09	0.081	659.35	50.00
512	−20	MMD, $\Gamma_{\Theta_{h}}=0$	248	−17.38	0.076	659.35	51.56
512	−20	MMD, $\Gamma_{\tau }=0$	250	−19.81	0.080	659.35	51.17
512	−20	MMD	250	−19.81	0.080	659.35	51.17
64	-	Fully populated	64	−13.26	0.614	81.29	0
64	−20	MMD	32	−13.28	0.687	80.00	50.00
64	−15	MMD, $\Gamma_{SLL}=0$	36	−15.05	0.621	80.00	43.75
64	−15	MMD, $\Gamma_{\Theta_{h}}=0$	32	−14.08	0.608	80.00	50.00
64	−15	MMD, $\Gamma_{\tau }=0$	32	−14.43	0.633	80.00	50.00
64	−15	MMD	32	−14.20	0.616	80.00	50.00

**Table 3:** *Comparative assessment* (P=N; I=1000)—pattern features.

Solution	Array Type	τ	SLL	$\Theta_{h}$	*L*	ΔN
			[dB]	[deg]	[*λ*]	[%]
[13]	Fully populated	256	−13.26	0.154	329.03	0
This work	Thinned	129	−18.29	0.159	329.03	49.61
[2]	Fully populated	512	−13.26	0.094	542.90	0
This work	Thinned	257	−19.97	0.097	542.90	49.80
[31]	Sparse	64	−13.00	0.250	206.45	37.25
This work	Thinned	64	−16.08	0.241	206.45	37.25

**Table 4:** *Robustness analysis* (non-ideal radiating elements)—features of far-field projections of WGA at different wavelengths.

λ	*f*	$L_{\mathrm{WGA}}$	$\theta_{0\left|y\right.}$	$\Theta_{h\left|y\right.}$	SLL	${\tilde{\beta }}_{-1}$	κ
[nm]	[THz]	[λ]	[deg]	[deg]	[dB]	[nm^−1^]	[nm^−1^]
1500	200.00	32.20	−3.36	1.55	−13.01	$-2.46\times 10^{-4}$	$1.29\times 10^{-7}$
1520	197.37	31.78	−5.74	1.58	−12.99	$-4.13\times 10^{-4}$	$1.51\times 10^{-7}$
1540	194.81	31.36	−8.16	1.61	−12.97	$-5.79\times 10^{-4}$	$1.74\times 10^{-7}$
1560	192.31	30.96	−10.56	1.64	−12.93	$-7.38\times 10^{-4}$	$1.94\times 10^{-7}$
1580	189.87	30.57	−12.98	1.68	−12.88	$-8.93\times 10^{-4}$	$2.13\times 10^{-7}$
1600	187.50	30.19	−15.43	1.72	−12.87	$-1.05\times 10^{-3}$	$2.23\times 10^{-7}$

## Data Availability

Data are contained within the article.
